# Concurrent herbivory and metal accumulation: The outcome for plants and herbivores

**DOI:** 10.1002/pei3.10088

**Published:** 2022-09-06

**Authors:** Diogo P. Godinho, Helena C. Serrano, Sara Magalhães, Cristina Branquinho

**Affiliations:** ^1^ Centre for Ecology, Evolution and Environmental Changes (cE3c) Faculdade de Ciências da Universidade de Lisboa Lisbon Portugal; ^2^ Departamento de Biologia Animal Faculdade de Ciências da Universidade de Lisboa Lisbon Portugal; ^3^ Departamento de Biologia Vegetal Faculdade de Ciências da Universidade de Lisboa Lisbon Portugal

**Keywords:** abiotic stress, biotic stress, elemental defense, spectral reflectance, spider mites

## Abstract

The effects of metals on plants and herbivores, as well as the interaction among the latter, are well documented. However, the effects of simultaneous herbivory and metal accumulation remain poorly studied. Here, we shed light on this topic by infesting cadmium‐accumulating tomato plants (*Solanum lycopersicum*), either exposed to cadmium or not, with herbivorous spider mites, *Tetranychus urticae* or *T. evansi* during 14 days. Whereas on plants without cadmium *T. evansi* had higher growth rate than *T. urticae*, on plants with cadmium both mite species had similar growth rates, which were lower than on plants without metal. Plants were affected by both cadmium toxicity and by herbivory, as shown by leaf reflectance, but not on the same wavelengths. Moreover, changes in leaf reflectance on the wavelength affected by herbivores were similar on plants with and without cadmium, and vice versa. Long‐term effects of cadmium and herbivory did not affect H_2_O_2_ concentrations in the plant. Finally, plants infested with spider mites did not accumulate more cadmium, suggesting that metal accumulation is not induced by herbivory. We thus conclude that cadmium accumulation affects two congeneric herbivore species differently and that the effects of herbivory and cadmium toxicity on plants may be disentangled, via leaf reflectance, even during simultaneous exposure.

## Graphical abstract

Infestation with spider mites does not lead to higher cadmium accumulation in tomato plants, despite this metal negatively affecting the herbivores. Simultaneous exposure to metals and herbivores leads to distinct effects on plant physiology that can be detected through leaf spectral reflectance.

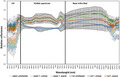

## 1 INTRODUCTION

Plants are often simultaneously exposed to several abiotic and biotic stresses. Their response to combinations of different stresses is well described, both at a physiological and at a molecular level (Ben et al., 2014; Guo et al., 2016; Havko et al., 2020; Nguyen et al., 2016; Pandey et al., 2015; Weldegergis et al., 2015; Ximénez‐Embún et al., 2016; Ximénez‐Embún et al., 2017). For example, drought increases the accumulation of phytohormones such as jasmonic acid and abscisic acid, hampering subsequent herbivore infestations (Nguyen et al., 2016) and herbivory decreases plant responses to heat stress by stimulating stomatal closure (Havko et al., 2020). However, most experiments (in controlled conditions) have exposed plants to abiotic and biotic stresses sequentially (Guo et al., 2016; Nguyen et al., 2016; Weldegergis et al., 2015). This design may underestimate the potential interaction among biotic and abiotic stresses.

One particular abiotic stress that plants experience is metal toxicity, occurring in geochemically specific areas or in areas of anthropogenic disturbance, where bioavailable metal concentrations in the soil are high. Some plants respond to this stress by taking up the metal from the soil and accumulating it into their shoots, in amounts that are toxic to most organisms, that is, hyperaccumulation (Baker, 1987). This may serve as defense against herbivores (Martens & Boyd, 1994). Indeed, many studies have shown negative effects of metal accumulation on herbivores, and that herbivores are repelled by high concentrations of metal in controlled experiments (Boyd & Moar, 1999; Freeman et al., 2006; Hanson et al., 2003; Jhee et al., 2005; Martens & Boyd, 1994; Quinn et al., 2010). Under natural conditions, metal accumulating plants suffer less herbivory than neighboring non‐accumulators, which provides further evidence for the effectiveness of this defense (Freeman et al., 2007; Galeas et al., 2008; Kazemi‐Dinan et al., 2014; Martens & Boyd, 2002; Rathinasabapathi et al., 2007).

Even though metal hyperaccumulating plants are more tolerant to metal toxicity than their non‐accumulating counterparts (Maestri et al., 2010), increasing the amounts of metals accumulated may take plants to a threshold above which the negative effects of metal accumulation are higher than the positive ones (Boyd, 2012). Therefore, it might be advantageous to accumulate high amounts of metal only when these provide an advantage, reducing herbivory. This may select for herbivore‐induced metal accumulation, rather than for its constitutive expression. However, this hypothesis can only be tested if metal accumulation is possible during herbivore infestation, and not limited to the plant growth period previous to herbivore infestation. In contrast with the vast knowledge on the induction of organic plant defenses by herbivory, only two studies have addressed whether plants accumulate more metal when facing herbivory (Plaza et al., 2015; Stolpe et al., 2017). Indeed, *Arabidopsis halleri* was shown to accumulate more cadmium in its leaves when exposed to herbivory by *Pieris rapae* (Plaza et al., 2015) and in the phloem when exposed to aphid herbivory (Stolpe et al., 2017). Still, it is unclear if metal accumulation, as an active defense mechanism, can be generalized to other metal‐accumulating plant species and other herbivores.

Most methods to analyze the response of plants to stress are destructive, limiting the number of analyses performed in a plant and hampering the obtention of repeated measures over time and of values for different traits for the same plant. One way to circumvent this set‐back is to use non‐destructive methods like the analysis of the multispectral reflectance of leaves. This technique is non‐invasive, allowing for the same plant to be measured at several points in time (Carter, 1993; Carter & Knapp, 2001), which is ideal to characterize traits of the same plant, before and after exposure to a given stress. The analysis of leaf reflectance may be used to characterize the plant physiological state, providing information regarding the normalized difference vegetation index (NDVI), water content, chlorophyll content, among other traits (Fabre et al., 2010; Jones et al., 2007). Changes in reflectance in the UV region, are associated with differences in leaf texture (e.g., rugosity, glandular trichomes) and with the production of reactive oxygen species (ROS) and secondary compounds in response to metal toxicity (Carter, 1993; Carter & Knapp, 2001; Grant et al., 2003). Additionally, the destructive effects of herbivory on the photosynthetic apparatus are associated with changes in the reflectance of visible and near infra‐red wavelengths (Luedeling et al., 2009; Martin et al., 2015; Peñuelas et al., 1995).

Changes in the concentration of reactive oxygen species are involved in the initial steps of the response of plants to both abiotic and biotic stresses, with the production and metabolization of such compounds being well described, as well as the signals triggering this production (Mittler, 2002). However, in the long term, the involvement of ROS in response to abiotic and biotic stresses is conflicting (Mittler, 2002). Whereas abiotic stress induces the production of ROS‐scavenging enzymes, which reduce the amount of ROS over time, biotic stress may suppress the activity of these enzymes, leading to an over‐accumulation of ROS (Mittler, 2002). Despite this conflict, little is known about how plants manage ROS production and scavenging when exposed to both stresses simultaneously.

Here we address the above‐mentioned gaps in the knowledge of the simultaneous response of plants to metals and herbivory, using a system composed of tomato plants (*Solanum lycopersicum*), which accumulate cadmium in their leaves, and two species of herbivorous spider mites, *Tetranychus urticae* and *T. evansi*. Previously, we exposed tomato plants sequentially to cadmium stress and herbivory by spider mites (Godinho et al., 2018). We found that exposure to up to 1.5 mM of cadmium chloride resulted in no differences in growth and biomass of tomato plants and led to cadmium accumulation on the leaves up to 150 (mg/kg), which is consistent with this variety being a hyperaccumulator of this metal (Godinho et al., 2018). Still, we observed changes in the UV‐B reflectance of tomato plants exposed to cadmium, possibly associated with production of phenolic compounds in response to this stress (Godinho et al., 2018). Additionally, spider mite herbivory is associated with changes in reflectance in the visible wavelengths (Luedeling et al., 2009). However, how simultaneous exposure to metal toxicity and herbivory affects leaf reflectance is unknown. The effect of double exposure on leaf reflectance may depend on whether the responses to each stress are synergistic or antagonistic and/or whether the response to one stress is stronger. Moreover, cadmium accumulation by tomato plants had a hormetic effect on the oviposition rate of both species of spider mites, which increased with mild concentrations of cadmium but decreased at higher concentrations (Godinho et al., 2018). This response of spider mites to cadmium accumulation in tomato plants was driven by the metal itself, rather than by metabolic changes in the accumulating plants (Godinho et al., 2022). Thus, we hypothesize that, when exposed simultaneously to mild concentrations of cadmium and spider mite herbivory, tomato plants may increase cadmium accumulation, to hamper herbivore performance. Also, the two spider mite species interact differently with tomato plants: while *T. urticae* infestation triggers the induction of plant organic anti‐herbivore defenses, infestation by *T. evansi* suppresses those defenses (Godinho et al., 2016; Kant et al., 2004; Sarmento, Lemos, Bleeker, et al., 2011). This pattern of induction or suppression was unaffected by plant cadmium accumulation, when spider mites were infesting plants after that accumulation (Godinho et al., 2018). Still, given that both species presented a similar response to cadmium, we hypothesize that none is able to suppress the accumulation of such metal in tomato plants. Here, by using a full orthogonal design, with plants exposed to cadmium, to spider mites or both stressors simultaneously, we aimed to shed light on the joint effects of metal toxicity and herbivory on plants.

## 2 MATERIAL AND METHODS

### 2.1 Biological material

Tomato plants (*Solanum lycopersicum*, var. Moneymaker) were sown in a climate chamber (25 °C, photoperiod 16/8 h light/darkness), in a soil/vermiculite mixture (4:1), being watered thrice a week with tap water, for 2 weeks. After this period, plants were watered twice a week, with 60 ml of either distilled water (un‐exposed) or a cadmium chloride solution (0.5 mM), for two and a half more weeks, which consisted in five different watering moments. All plants were watered an additional time per week, with tap water, to compensate for micronutrients deficiencies.

The populations of spider mites used in this study (2018–2019) were collected nearby Lisbon, Portugal: *Tetranychus urticae* in 2010 (Clemente et al., 2016) and *T. evansi* in 2013 (Zélé et al., 2018). Both species were maintained in plastic cages containing entire tomato plants as described in Godinho et al., 2018. From these populations, cohorts of spider mites were prepared by isolating groups of 75 females on tomato leaves, which were placed on wet cotton wool in Petri dishes. These females laid eggs for 48 h, being removed afterwards. The daughters of these females were used in the subsequent experiments 14 days later.

### 2.2 General methodology

The experimental assays used 4.5‐week‐old plants. The spectral reflectance of the fourth oldest leaf of each plant was measured using a UniSpec spectroradiometer (PP Systems), and replicated on five different leaflets of each plant. The spectral data generated by these measurements were determined by calculating spectral reflectance factors (R) for each wavelength (between 300.4 and 1148.1 nm with intervals of 3.4 nm). These factors were obtained by normalizing the reflected radiation from the leaves, in the beginning of each assay, with a reflectance white standard, following manufacturer instructions.

Afterwards, the same leaf (of plants with or without cadmium) was infested, using a fine brush, with 100 mated females (20 on each of five different leaflets of the same leaf) of either *T. urticae* or *T. evansi*, or it was left un‐infested, resulting in six treatments (*N* = 12 plants per treatment). The infestation with spider mites lasted 14 days, which roughly corresponds to the generation time of these species, then the spectral reflectance was measured again. By measuring each plant at two different time points, before and after exposure to cadmium, exposure to spider mites or exposure to both, we obtained the response of each individual plant to each of these stressors. During the exposure period, plants were maintained in the same conditions (i.e., 25°C, photoperiod 16/8 h light/darkness) and exposed to the same watering conditions as before, ensuring the bioavailability of cadmium for plants during infestation. Subsequently, the number of adult females (the offspring of the infesting females) was registered for each plant. Note that the infested leaf was isolated with lanolin in the petiole, to prevent mites from moving to other leaves, but mites could move freely among abaxial and adaxial surfaces of all leaflets of the same leaf.

To quantify the amount of cadmium accumulated on the experimental leaves, spider mites, web, and eggs were removed (when present) with a makeup brush and the leaf was dried at 60°C, until constant mass, and ground. Cadmium was then quantified using inductively coupled plasma–atomic emission spectrometry (ICP–AES; LAIST), after nitric acid digestion, with a detection limit of 1 mg/kg.

Additionally, fresh leaf material was frozen at −80°C, in liquid nitrogen to quantify ROS (in the form of H_2_O_2_). For the extraction, frozen leaf material was weighted (~100 mg tissue/0.5 ml Phosphate–Citrate Buffer, pH 5.4) and ground in liquid nitrogen (Antioxidant Assay Kit CS0790, Sigma‐Aldrich). The macerate was placed in microtubes (2 ml), centrifuged and the supernatant used to determine the concentration of ROS using a modification of the assay protocol of the manufacturer (Antioxidant Assay Kit CS0790, Sigma‐Aldrich). The concentration of H_2_O_2_ in the supernatant was determined through changes in absorbance, using a mixture of 50 μl extract in Assay buffer, 20 μl myoglobin working solution, and 150 μl ABTS substrate solution (2,2′‐azino‐bis[3‐ethylbenzthiazoline‐6‐sulfonic acid] with Phosphate–Citrate buffer). Values were then standardized with an H_2_O_2_ calibration curve prepared using 50 μl Assay buffer, 20 μl myoglobin working solution, and 0, 25, 50, 75, or 150 μl ABTS with 3.25 mM H_2_O_2_ (total volume 150 μl, diluted with ABTS without H_2_O_2_). Absorbance was measured for all reactions at 405 nm in an Epoch 2 Microplate Photometer (BioTek, BioSPX®).

The assays were done in four blocks, separated in time, each consisting of three replicate plants per experimental treatment.

### 2.3 Statistical analyses

The number of female mites alive on the plant after 14 days was compared using a generalized linear mixed model (*glmer*) with a Poisson distribution. Exposure to cadmium, infesting species (*T. urticae* or *T. evansi*) and their interaction were used as fixed factors and block as a random effect. Because there was a significant interaction between the infesting species and exposure to cadmium, comparisons among treatments within each factor were performed using the *phia* package in R (de Rosario‐Martinez, 2015).

The amount of cadmium (mg per kg of sample) and the amount of H_2_O_2_ (μmol per gram of sample) present in the sampled leaf of each plant were compared between treatments using general linear mixed models (*lmer*) with a Gaussian distribution. Exposure to cadmium, infestation (infested with *T. urticae*, infested with *T. evansi* or uninfested), and their interaction were used as fixed factors and block as a random effect.

The effect of spider mite infestation and exposure to cadmium, on the spectral reflectance of leaves of tomato plants, was analyzed using a multivariate analysis of variance with distance matrices (*Adonis* function, *vegan* package; [Oksanen et al., 2015]). For each plant, the difference in reflectance, for each wavelength (between 300.4 and 1148.1 nm with intervals of 3.4 nm), was determined between the beginning and the end of the experiment and was used as the response variable. Spider mite infestation, the cadmium concentration supplied, and their interaction, were used as fixed factors. Since there was a significant effect of spider mite infestation on leaf reflectance and because the Adonis function does not support post hoc contrasts, we compared the effect of the two spider‐mite species using the same model but using a subset of the data excluding uninfested plants. Because cadmium accumulation was shown to affect the reflectance of tomato plants at the UV wavelengths (Godinho et al., 2018), we repeated the analysis using only the reflectance factors of wavelengths between 300.4 and 395 nm. Changes in the visible spectra (400–700 nm) have been used to detect the damage of spider mites on several plant species (Luedeling et al., 2009; Martin et al., 2015; Peñuelas et al., 1995). Additionally, leaf chemistry can be determined through the reflectance in the near infrared region of the spectrum (780–1400 nm), this method being used to assess the intensity of herbivory (Foley, 2009; Gillon et al., 1999; Meuret et al., 1993). Therefore, we also analyzed changes in reflectance within those wavelengths. Due to technical issues, the reflectance of plants of the last block could not be measured at both time points, thus we excluded them from the analysis, including only nine plants per treatment (from the three other blocks).

## 3 RESULTS

**FIGURE 1:**
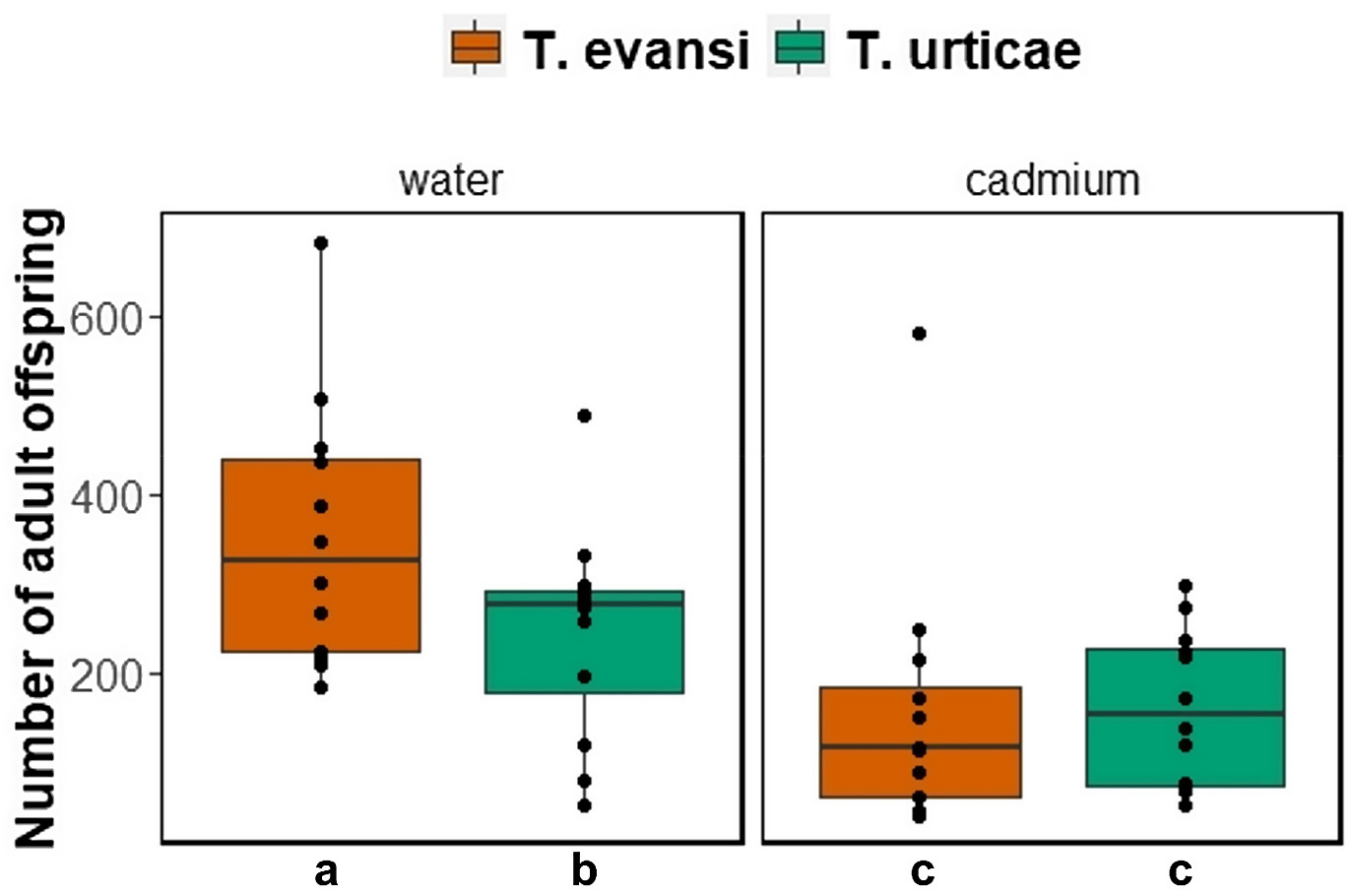
Number of adult offspring (*T. evansi* ‐ orange; *T. urticae* ‐ green) on tomato plants after 14 days of infestation (*N* = 12 plants per treatment). Plants were watered with 0.5 mM of cadmium chloride (right panel) or with distilled water (left panel), twice a week. Letters represent significant differences in the a posteriori contrasts (*p* < .05).

The number of mites of each species after 14 days on tomato plants was differently affected by cadmium accumulation (Figure 1; interaction between cadmium exposure and infesting species: χ^2^_1_ = 97.01; *p* < .001). Indeed, *T. evansi* was more affected by cadmium than *T. urticae*: on plants without cadmium, the number of *T. evansi* was higher than that of *T. urticae* (Figure 1; χ^2^_1_ = 235.68, *p* < .001) but this was not the case on plants with cadmium (Figure 1; χ^2^_1_ = 0.55, *p* = .46). Still, the number of mites was significantly lower on plants exposed to cadmium than on un‐exposed plants, both for *T. urticae* (Figure 1; χ^2^_1_ = 842.28, *p* < .001) and for *T. evansi* (Figure 1; χ^2^_1_ = 192.2, *p* < .001).

**FIGURE 2:**
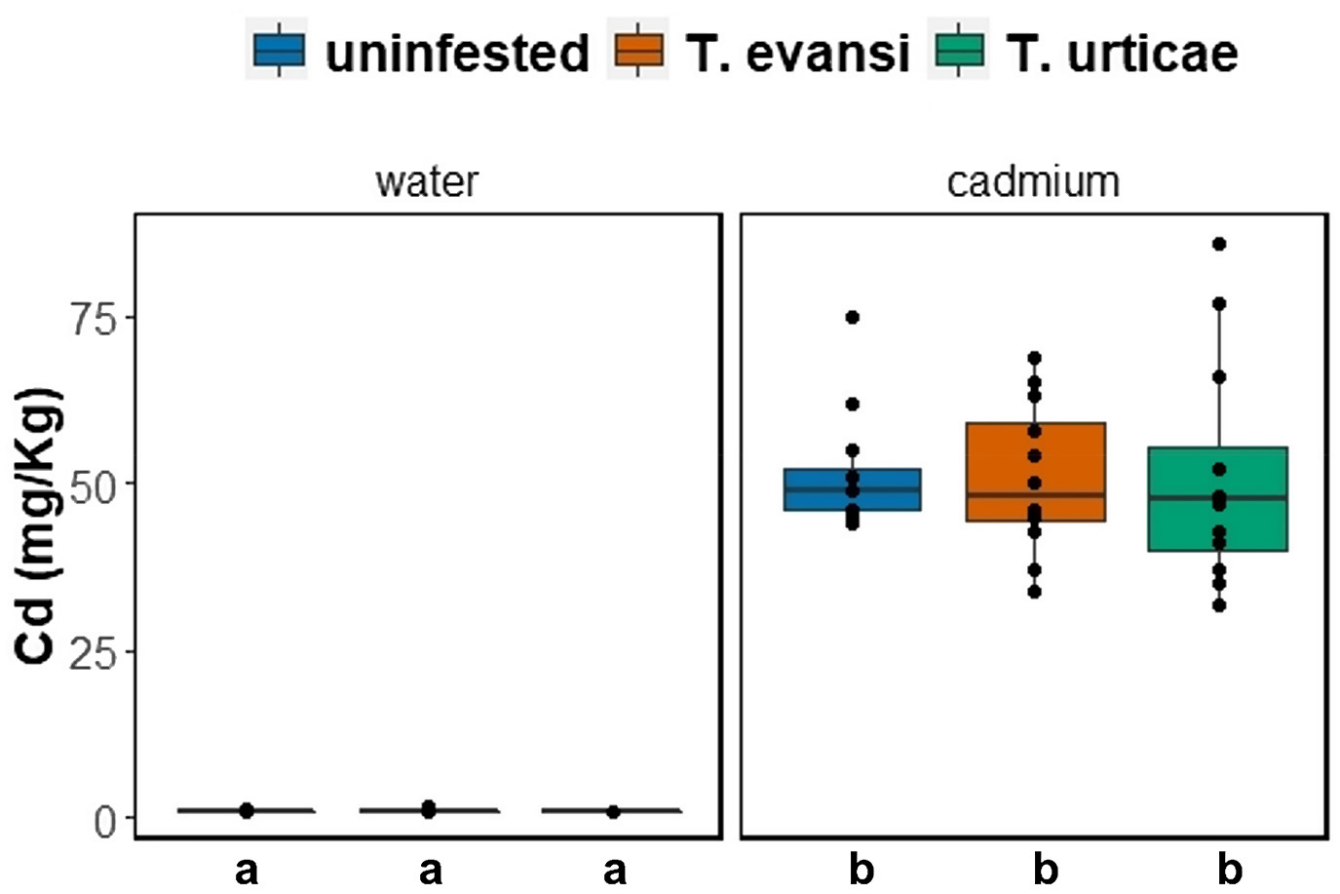
Accumulation of cadmium (*N* = 12 plants per treatment) on leaves of plants exposed to 0.5 mM of cadmium chloride (right panel, “cadmium”) or not (left panel, “water”). Furthermore, leaves were either un‐infested (blue) or infested with *T. evansi* (orange) or *T. urticae* (green) for 2 weeks. Letters represent significant differences (*p* < .05).

Plants exposed to cadmium accumulated higher amounts of this metal on their leaves (≈50 mg Cd/kg) than plants that were not exposed to this metal (<1 mg Cd/kg; Figure 2; F_1_,_65_ = 648.16; *p* < .001). However, this accumulation was unaffected by plant infestation with spider mites (interaction between cadmium exposure and spider mite infestation: Figure 2, F_2,65_ = 0.06, *p* = .94).

**FIGURE 3:**
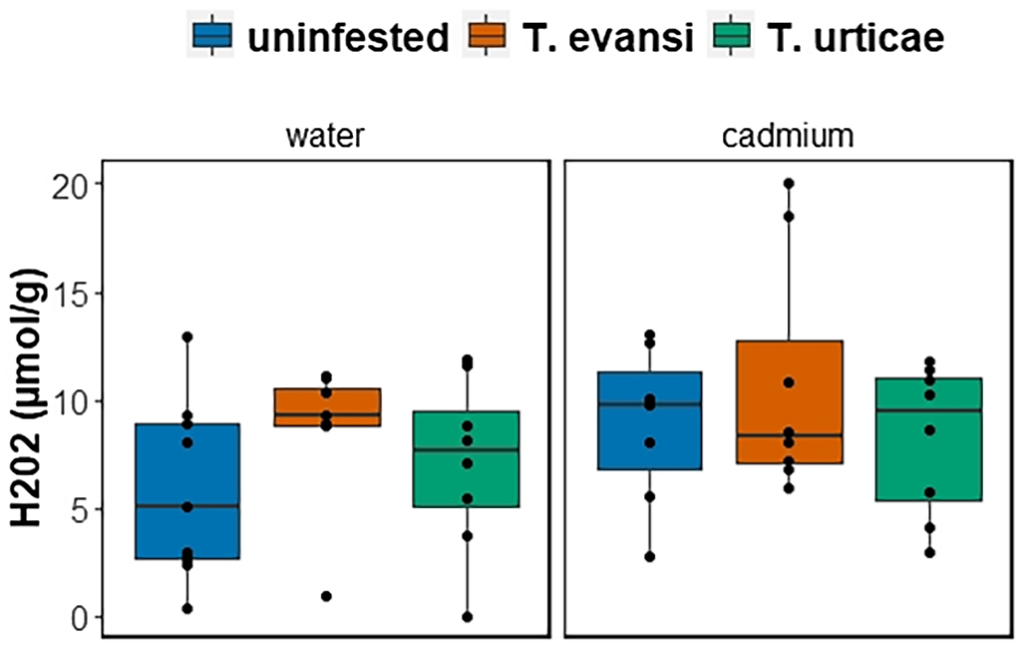
Accumulation of H_2_O_2_ (*N* = 12 plants per treatment) on leaves of plants exposed to 0.5 mM of cadmium chloride (right panel, “cadmium”) or not (left panel, “water”). Furthermore, leaves were either un‐infested (blue) or infested with *T. evansi* (orange) or *T. urticae* (green), for 2 weeks. There was no significant effect of either cadmium exposure or infestation by spider mites on the amount of H_2_O_2_ accumulated after 14 days in the plant.

The amount of H_2_O_2_ (μmol per gram of sample) present in tomato leaves was not affected by cadmium exposure after 15 days (Figure 3; F_1,41_ = 0.97, *p* = .933), spider mite infestation (Figure 3; F_2,41_ = 1.12, *p* = .31) nor by the interaction between these two stressors (Figure 3; F_2,39_ = 0.14; *p* = .87).

**FIGURE 4:**
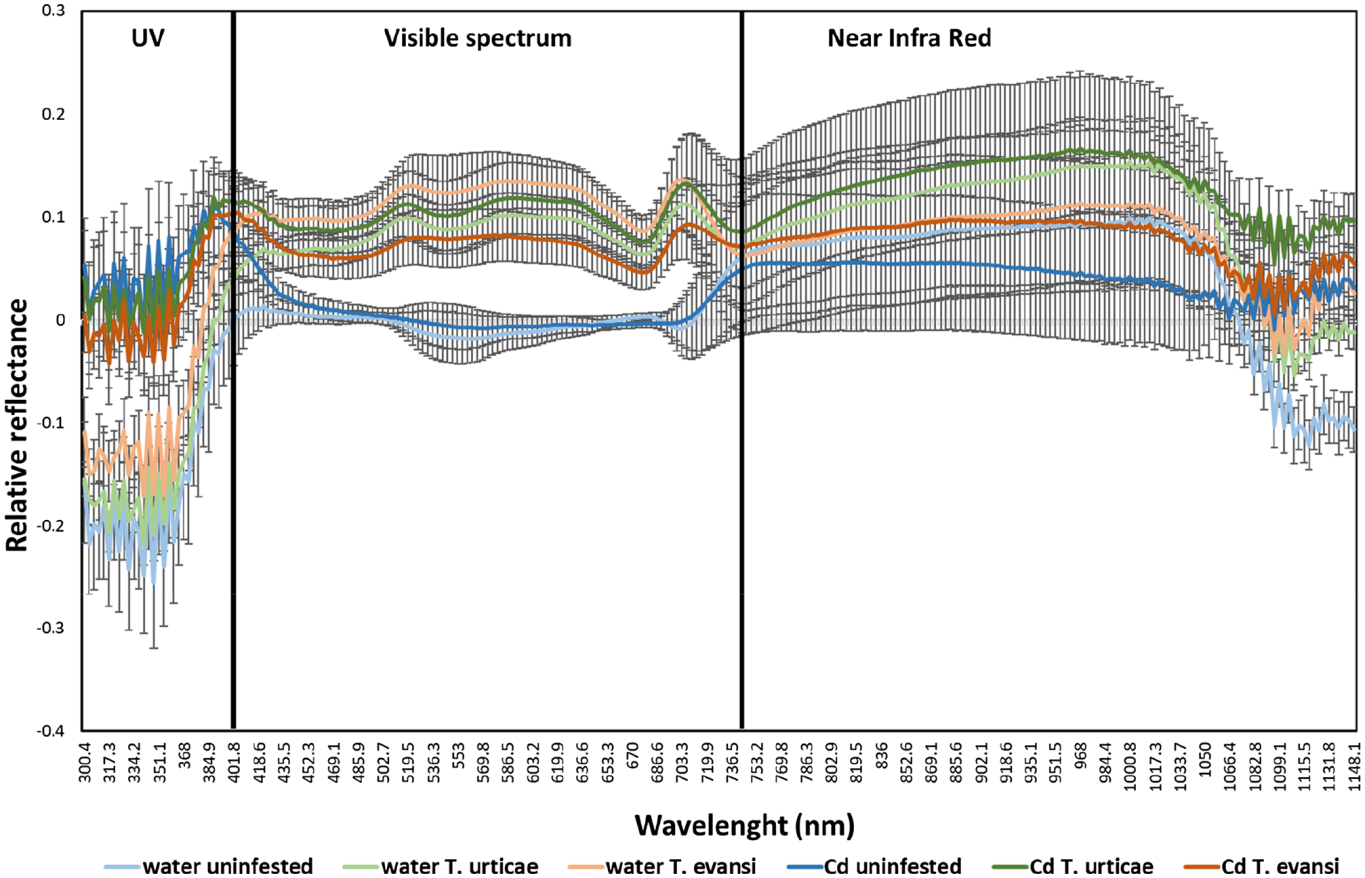
Average difference in leaf spectral reflectance (wavelengths between 300.4 and 1148.1 nm; ±SE, *N* = 9 plants per treatment) between the beginning and the end of the experiment. During the trial, plants were exposed to 0.5 mM of cadmium chloride (“Cd”), or not (“water”), and they were either un‐infested (blue) or infested with 100 *T. evansi* (orange) or 100 *T. urticae* mated females (green).

In the UV area (300–395 nm), exposure to cadmium had a significant effect on plant reflectance (F_1,50_ = 24.51; *p* < .001; Figure 4), both on plants infested by spider mites and on uninfested plants (interaction between infestation and cadmium: F_2,48_ = 1.41; *p* = .24). Infestation by spider mites had no effect on the reflectance at these wavelengths (F_2,50_ = 0.52; *p* = .61; Figure 4).

In the visible wavelength spectrum (400–700 nm), exposure to cadmium had no effect on plant reflectance (F_1,50_ = 0.36; *p* = .57; Figure 4) but infestation by spider mites did (F_2,50_ = 14.68; *p* < .001; Figure 4), both on plants exposed to cadmium and on plants unexposed to this metal (interaction between infestation and cadmium: F_2,48_ = 1.65; *p* = .20). The reflectance on the visible wavelength spectrum was similarly affected by *T. urticae* and by *T. evansi* (F_1,34_ = 0.06; *p* = .88; Figure 4).

Finally, for the spectral reflectance on near infrared wavelengths (780–1400 nm), there was no effect of either infestation with spider mites (F_1,50_ = 1.06; *p* = .36; Figure 4) or exposure to cadmium (F_1,50_ = 0.69; *p* = .42; Figure 4).

## 4 DISCUSSION

In this study, we tackled underexplored aspects of plant–herbivore interactions in metal polluted environments by assessing the simultaneous exposure of plants to biotic and abiotic stressors. First, we show that exposure to cadmium‐accumulating plants for one generation reduces the fitness differences between *T. evansi* and its congeneric *T. urticae*, that is, differences in the number of adult offspring produced (Young & Wrensch, 1982). Thus, metal accumulation may act as an equalizing mechanism affecting the coexistence of herbivore species on metal polluted environments (Chesson, 2000; HilleRisLambers et al., 2012). Second, we show that the levels of ROS on plants after 14 days were not affected by herbivory nor by cadmium exposure, suggesting that plants had regain their homeostatic balance after an initial response to those stressors. In contrast, the spectral reflectance of tomato leaves reveals that plants were affected by herbivory and cadmium exposure, but at different wavelengths, suggesting that these biotic and abiotic stresses acted on different plant traits. Despite these effects, herbivory did not induce further cadmium accumulation on tomato plants.

*Tetranychus evansi* is a specialist of Solanaceae plants, being its reproductive performance on tomato plants generally higher than that of other spider mites, including *T. urticae* (Godinho et al., 2016, 2018; Paulo et al., 2018; Schimmel et al., 2017), leading to the exclusion of *T. urticae* on tomato plants with *T. evansi* (Sarmento, Lemos, Dias, et al., 2011). Here, we recapitulate this result, but we also show that this reproductive advantage is lost when tomato plants are exposed to cadmium. Previously, we have shown that induction and suppression of tomato defenses, by *T. urticae* and *T. evansi*, respectively, are not affected by cadmium accumulation (Godinho et al., 2018). Thus, the similar performance of *T. evansi* and *T. urticae* on tomato plants exposed to cadmium suggests that the effect of this metal overrules that of plant defenses. This is in agreement with previous results showing that the direct effect of cadmium on spider mites is stronger than those stemming from physiological changes in the plant (Godinho et al., 2022). Another possibility is that when plants are exposed to both stresses simultaneously (cadmium toxicity and herbivory) there is an interaction between the two defense mechanisms that was not observed when the stresses were sequential. If suppression of plant defenses is costly to *T. evansi*, there may be a trade‐off with the mechanisms to cope with cadmium toxicity, leading to this spider mite being more affected than *T. urticae*. If this is the case, suppression of organic plant defenses may be counter‐selected in metal polluted environments, a hypothesis waiting to be tested. Alternatively, *T. urticae* may have a higher metal detoxifying ability, being less affected than *T. evansi* by longer exposure to cadmium.

We have previously shown that, under controlled conditions, spider mites had a hormetic response to cadmium accumulation in tomato plants, having higher oviposition rates at intermediate cadmium concentrations (Godinho et al., 2018). Here, we do not recapitulate this result. The differences observed in the response of spider mites to cadmium could be a result of (i) plants having been exposed to both cadmium and spider mites simultaneously, and/or (ii) the longer exposure period of spider mites to cadmium in this experiment (14 days) compared to the previous (4 days). Indeed, the longer duration of the current experiment might have resulted in plants accumulating more cadmium than before, over time, surpassing the level of beneficial concentrations to the spider mites. However, the amount of cadmium accumulated in the leaves in this study is within the levels that resulted in a positive effect in spider mites in our previous study (Godinho et al., 2018). Therefore, the decreased performance of spider mites in the current study is likely due to their continuous exposure to plants with cadmium, reaching toxic concentrations beyond the hormetic effect. This may have important ecological and evolutionary consequences. Indeed, if the environment is heterogeneous, spider mites may alternate between eating plants with metals for a short period and plants without metals, increasing in this way their overall fitness. In contrast, in environments, only with plants accumulating metals, the fitness of spider mites is overall reduced, even for mild metal concentrations. Alternatively, cadmium concentration might affect differentially different stages of development of spider mites. Indeed, the previous study showing a hormetic effect was done on adults (Godinho et al., 2018), whereas here our measure includes the survival of juvenile stages. These two hypotheses are non‐exclusive. Disentangling between them could shed light on the mechanisms of the effect of metals on herbivores.

We show that tomato plants did not accumulate more cadmium when exposed to herbivory. This suggests that metal accumulation in these plants is not inducible by spider mite infestation. This result contrasts with what was found for *Arabidopsis halleri*, which increases the accumulation of cadmium and zinc in the leaves and in the phloem, as a response to herbivory (Plaza et al., 2015; Stolpe et al., 2017). However, herbivory on *A. thaliana*, did not induce cadmium uptake (Plaza et al., 2015), suggesting that this could be a plant‐species specific trait. One possibility is that tomato plants are more sensitive to cadmium than *A. halleri* and in this case, the accumulation of cadmium could reach a threshold above which there is toxicity for tomato plants. However, the amount of cadmium accumulated by the plants in this study, even though being above the hyper‐accumulation threshold (Pollard, 2000), was within the values that did not affect growth rate and biomass production of tomato plants (Godinho et al., 2018), suggesting little or no costs at this concentration. Another hypothesis is that the amount of cadmium accumulated by the plants was sufficient to diminish the effects of herbivory, hence there was no need for the plant to induce more metal uptake.

The latter hypothesis is in agreement with the fact that we did not find differences in ROS accumulation among plants in the long term. Neither cadmium toxicity, herbivory, nor the combination of both led to an increase in ROS accumulation. This suggests that either the plants were not stressed by these factors or, that the important role of ROS mediating the early response of plants to spider mites (Santamaría et al., 2012, 2018) and cadmium (Gratão et al., 2015; Mishra et al., 2014) is not swayed through time, possibly because plants recovered from the initial response to those stressors and regained their homeostatic balance.

In contrast, leaf reflectance shows that plants were affected both by cadmium and by spider mite infestation, and these effects can be disentangled even when they occur simultaneously because they affected different components of the leaf spectrum. Cadmium exposure led to changes in reflectance in the UV region, mainly associated with secondary metabolism, indicating effects either in leaf texture (e.g., rugosity, glandular trichomes) and/or in chemical molecules (e.g., waxes, secondary compounds) produced by the plant in response to cadmium accumulation (Carter, 1993; Carter & Knapp, 2001; Grant et al., 2003). In contrast, leaf reflectance was broadly affected by herbivory, in particular in the visible spectrum (400–700 nm), more related with primary metabolism, which is indicative of damage on the photosynthetic apparatus (Luedeling et al., 2009; Martin et al., 2015; Peñuelas et al., 1995). These effects of spider mite infestation on spectral reflectance were similar among plants exposed or not to cadmium. Interestingly, the effects of cadmium accumulation on UV reflectance were also not affected by spider mite infestation. Heavy metal stress and pathogen infestation have been shown to affect the water content and leaf structure, which is associated with changes in the reflectance at near infra‐red (NIR) wavelengths (Chi et al., 2012; Thomas et al., 2018). Here, however, we did not observe such effects on NIR wavelengths, confirming that neither cadmium accumulation, nor spider mite infestation affected such plant traits, as previously observed in this system (Godinho et al., 2018). Still, the effects on spectral reflectance, both by cadmium exposure and by spider mite infestation, support the hypothesis that the plants were indeed stressed by these factors, but that cadmium exposure and spider mite infestation affect different plant traits, that is, different physiological mechanisms. These spectral reflectance results show that, even though the growth rate of spider mites was hampered by cadmium accumulation, plants were similarly affected by herbivory, independently of being exposed to cadmium or not. Thus, female adults introduced on the plant were able to damage cadmium exposed plants as much as un‐exposed plants during the 14‐day period in which they were infesting the plant, suggesting minor negative effects of cadmium on the spider mites. Despite this, the growth rate of spider mites was hampered in cadmium exposed plants, therefore, these results advocate the hypothesis that juvenile stages are more susceptible to cadmium than adult spider mites. In any case, our results show that spectral reflectance is a useful tool to disentangle between the effects of different stressors even when these occur simultaneously and affect different plant components.

In conclusion, we show that cadmium accumulation is not inducible by herbivory in tomato plants, being restricted to the bioavailable amounts of metals. Understanding whether metal accumulation is an inducible or a passive mechanism, in other plant–herbivore systems, may disclose key aspects of the ecology and evolution of metal accumulation as a defense against herbivory. We also show that metal accumulation may even out differences in fitness of herbivores in the same plant. As some herbivores are more affected by metal accumulation than others, this may influence differently their distributions among plants with and without the metal, which will have important eco‐evolutionary consequences for the competition and coexistence of those herbivores.

## CONFLICT OF INTEREST

The authors declare no conflict of interest.

## Data Availability

Data are deposited in Figshare: https://doi.org/10.6084/m9.figshare.20473473.
